# GEnZ explorer: a tool for visualizing agroclimate to inform research and regulatory risk assessment

**DOI:** 10.1007/s11248-023-00354-w

**Published:** 2023-06-06

**Authors:** Rachel L. Melnick, Larissa Jarvis, Paul Hendley, Monica Garcia-Alonso, Marc J. Metzger, Navin Ramankutty, John L. Teem, Andrew Roberts

**Affiliations:** 1 Agriculture and Food Systems Institute, Washington, DC USA; 2 McGill University, 845 Sherbrooke Street West, Montréal, QC Canada; 3 Phasera Ltd., 7 Kenilworth Avenue, Bracknell, Berkshire UK; 4 Estel Consult Ltd., 5 Hillside Drive, Binfield, Berkshire UK; 5 School of Geosciences, Geography and the Lived Environment, The University of Edinburgh, Edinburgh, Scotland; 6 School of Public Policy and Global Affairs, The University of British Columbia, Vancouver, BC Canada; 7 Genetic Biocontrols LLC, Tallahassee, FL USA

**Keywords:** Agroclimatic zonation, Data transportability, Environmental risk assessment, Genetically engineered crops, Regulation

## Abstract

Confined field trials (CFT) of genetically engineered (GE) crops are used to generate data to inform environmental risk assessments (ERA). ERAs are required by regulatory authorities before novel GE crops can be released for cultivation. The transportability of CFT data to inform risk assessment in countries other than those where the CFT was conducted has been discussed previously in an analysis showing that the primary difference between CFT locations potentially impacting trial outcomes is the physical environment, particularly the agroclimate. This means that data from trials carried out in similar agroclimates could be considered relevant and sufficient to satisfy regulatory requirements for CFT data, irrespective of the country where the CFTs are conducted. This paper describes the development of an open-source tool to assist in determining the transportability of CFT data. This tool provides agroclimate together with overall crop production information to assist regulators and applicants in making informed choices on whether data from previous CFTs can inform an environmental risk assessment in a new country, as well as help developers determine optimal locations for planning future CFTs. The GEnZ Explorer is a freely available, thoroughly documented, and open-source tool that allows users to identify the agroclimate zones that are relevant for the production of 21 major crops and crop categories or to determine the agroclimatic zone at a specific location. This tool will help provide additional scientific justification for CFT data transportability, along with spatial visualization, to help ensure regulatory transparency.

**Supplementary Information:**

The online version contains supplementary material available at 10.1007/s11248-023-00354-w.

## Introduction

In most countries, the regulatory system for approving the cultivation of genetically engineered (GE) crops requires the preparation of an environmental risk assessment (ERA) to facilitate decision making. The first step in these ERAs is problem formulation, where the policy protection goals and the scope of the risk assessment are considered (Raybould 2006; Wolt et al. 2010; Sanvido et al. 2012; Garcia-Alonso and Raybould 2014). In this step, risk assessors compile all available relevant information to establish whether data requirements are fulfilled and if there is enough information to complete a risk characterization. For ERAs supporting applications for cultivation of GM crops, the information typically considered during the problem formulation step is gathered from various sources, such as peer reviewed scientific papers, scientific opinions generated by regulatory authorities, data generated to support the risk assessment for food and feed purposes, and data generated in other geographies. Following an analysis of these data, risk assessors can determine whether more data must be generated (analysis plan) or not. Therefore, the types of data generated to support ERAs depend on the data requirements set up by national regulation and the outcome of problem formulation.

Confined field trials (CFTs) are conducted to generate data in support of the ERA, predominantly agrophenotypic characterization of the GE plant. This data is used to assess the presence of any unintended, deleterious effects of the genetic modification. CFTs are designed to compare selected measurement endpoints between the GE plant and a suitable comparator grown under the same conditions. The trials are replicated spatially at multiple locations to establish whether environmental factors can influence the outcome. While there are no international standards for CFTs, they typically follow stringent standards for experimental design and similar (if not identical) measurement endpoints are used in trials regardless of where they take place (Garcia-Alonso et al. 2014; Nakai et al. 2015; Clawson et al. 2019). Due to the specific nature of these trials, they are carefully planned and managed beyond what is typical in a farmer's field to avoid confounding effects and sometimes, to limit access to the field (Roberts et al. 2014). The main distinction between CFTs of the same GE variety conducted at different locations is the agroclimate, as soil and growing conditions are optimally maintained regardless of location (Garcia-Alonso et al. 2014). As the management of CFTs limits variation of biotic and abiotic factors affecting the outcomes of the trials, there is likely little benefit to conducting CFTs for the same GE varieties every time a risk assessment is conducted in a new country.

Garcia-Alonso et al. (2014) published a conceptual framework for data transportability of the results of CFTs by addressing the practical requirements for the use of remotely generated field trial data to support ERAs and regulatory decision making for genetically engineered plants. This conceptual framework posits that there are four practical requirements for the use of remotely generated CFT data:

1. The CFTs must have been conducted, and data documented and reported, in a manner that meets minimum local regulatory requirements;
2. The environmental and agronomic conditions under which the CFT was conducted in the remote country(ies) must be relevant to the conditions in the local country where the GE event is intended to be cultivated;
3. The local regulator would need to be provided with an evidence-based justification for accepting data from CFTs conducted in the remote country(ies);
4. The local regulator would need a science-based process for identifying whether CFT data developed in one or more remote countries are sufficient to address local needs, or whether additional trials might be required.

While the concept of data transportability has been used or is proposed for use in regulatory risk assessment by several countries, such as Argentina, Canada, Japan, and the United States (Nakai et al. 2015; Matsushita et al. 2020; Vesprini et al. 2020), replacing local field trials with data transported from trials that were completed in another country has been difficult to implement in practice. It is also proposed that data transportability could benefit public sector developers who have limited funding to support replicating CFTs in each country, particularly those targeting subsistence farmers. Because of the comparative nature of agrophenotypic studies, which are typically conducted across diverse environmental conditions, Bachman et al. (2021) recently asserted that conclusions from well-designed studies that meet local requirements can be made independent of agroclimate or region, unless a risk-hypothesis opposes that consideration.

To support transportability of CFT data, an expert working group (EWG) was formed to identify open sources of agroclimate and crop production data that could be assembled to develop a tool to address points 2, 3 and 4 above, in order to facilitate data transportability. Ideally, this tool would be open source, freely available, and based on well-established technology to allow for expert consideration of data transportability in the regulatory process. The EWG identified the need to provide users with this tool in order to support them in deciding whether data generated in CFTs in one or more other countries are sufficient to address local regulatory needs and the need for regulatory authorities to cite a transparent scientific resource in their decision making.

For practical and scientific reasons, a generic, global, agroclimate zonation scheme was chosen to serve as the basis of the tool. In addition to allowing a single uniform representation of agroclimate that can be used across crops and geographies, a global scheme ensures that similar agroclimate zones in distant geographies are categorized correctly (Metzger et al. 2013a). Instead of trying to incorporate crop distribution and planting data into the agroclimate map, additional resources are provided to allow users to identify relevant agroclimates in the context of their particular crop or crops of interest. Several global agroclimate stratifications are available, and any of these could serve as the basis for such a tool (van Wart et al. 2013). Most of the available agroclimate zonation schemes are in reasonably good agreement as to global zonation. They differ in the number of zones, the transparency of the methodology used to generate them in their respective abilities, and the ease of accessing information underlying the zonation to build a tool around it (van Wart et al. 2013). The Global Environment Stratification System (GEnS) of Metzger et al. (2013b) was selected by the EWG based on both scientific and practical criteria detailed in Table 1.

**Table 1:** The scientific and practical criteria used to determine the applicability of GEnS zonation for developing a tool to identify surrogate CFT environments

Scientific criteria	Practical criteria
Peer reviewed, with demonstrated application for multiple purposes and regions	Transparent methodology
Enables a sufficient number of stratifications	Freely accessible
Data driven zonation that is not reliant on modeling or expert solicitation	Flexible, so the number of zones and strata can be adjusted for practical use
Statistically hierarchical	
Uses well accepted global data sets	
Validates against other zonation methods	
Sufficient spatial resolution	
Statistical method maximizes within-strata homogeneity	

GEnS was the first cluster methodology that established a global, climate-explicit zonation system. GEnS zonations are not specific to agriculture but have been used in other scientific studies where agroclimate is important, such as a study of hotspots of zoonotic diseases (Allen et al. 2017), to model the impact of climate change on biodiversity and rubber production in China (Zomer et al. 2014), and to map invasive amphibians and reptiles (Capinha et al. 2017) and the impact of climate on fungal community structure in top soil (Mukhtar et al. 2021). Beyond its acceptance by other researchers, the clustering methodology is open access, along with all data, and users could set the number of zones and strata.

Beyond agroclimatic zonation, information needed for the tool to be useful for considerations of data transportability include the geographic distribution of major crops and some method for understanding how important any particular agroclimate is for production of a specific crop. To meet this need, the EWG determined the open source agricultural statistics on land use gathered under Monfreda et al. (2008) to be an excellent source for cropping data, as they gathered agricultural census data from 206 countries, allowing users to determine net primary production by geographic distribution.

The goal of this work was to develop a tool that combined agroclimate zonation, specifically the GeNS of Metzger et al. (2013b), with the global land use data for agriculturally significant crops of Monfreda et al. (2008) to provide the information necessary to inform developers and risk assessors. This tool is useful for making decisions about the location of future CFTs or supporting the relevance of CFT data previously generated in other countries to a risk assessment that is being conducted. The objective of this manuscript is to document the open-source data and methods used to develop the tool and present case studies that demonstrate how the tool could be useful to the risk assessment process.

## Methods

### Software used for tool development

The data transportability tool was developed using the following open source software: Shiny for R Studio (https://shiny.rstudio.com/) and Leaflet (https://leafletjs.com/), a Javascript library for interactive maps. The tool is deployed online via shinyapps.io by RStudio. All pre-processing was done using the R Project for Statistical Computing (https://www.r-project.org/), QGIS (https://qgis.org), and MapTiler (https://www.maptiler.com/).

### Sources of data for the visualization tool

**Fig. 1:**
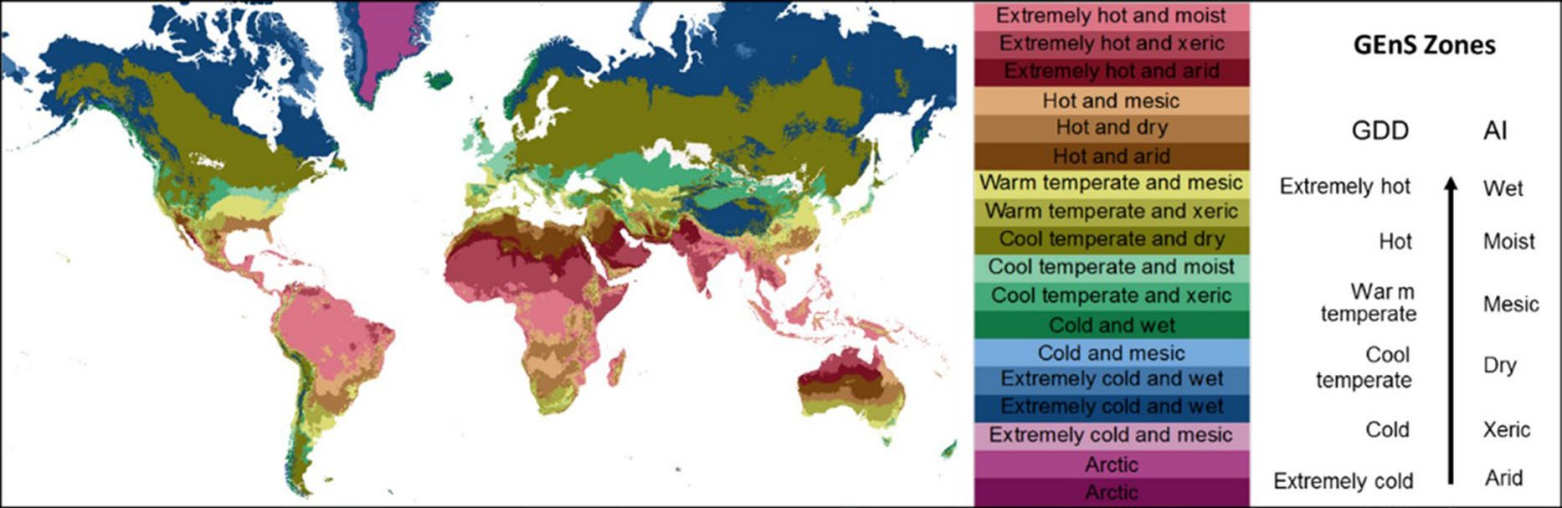
Global environnemental zones (GENZ). Color coding for zones is as indicated (and used in all subsequent figures). Agroclimatic zones vary primarily according to GDD (Growing Degree Days) and AI (Aridity Index)

To develop the tool, two sources of data were required, one to address agroclimate zonation and the other to address crop production by location. Metzger et al. (2013b) produced the Global Environmental Stratification (GEnS hereafter; available at https://doi.org/10.7488/ds/2354), a 30-arcsecond global climate classification generated through the statistical clustering of four pre-selected climate variables (growing degree days, aridity index, temperature seasonality, and potential evapotranspiration seasonality). The climate variables used for the statistical clustering were taken from Worldclim and CGIAR-CSI datasets. Clustering of agroclimate data resulted in 125 strata, which were then grouped into eighteen global environmental zones, or GEnZ (Fig. 1).

The GEnS zonation has been widely used for a variety of purposes in multiple locations and is well-documented within the peer-reviewed literature (van Wart et al. 2013; Zomer et al. 2014; Capinha et al. 2017; Mukhtar et al. 2021). Thus, GEnS zonation is a well-established approach that should not require further verification before it can be used by regulators or others interested in data transportability. Because the software is freely accessible, results can be readily reproduced and verified, further increasing the methodology’s transparency.

Monfreda et al. (2008) compiled global-scale, 5-min resolution data on crop-harvested area and yield for 175 crops by combining national and subnational crop statistics and global cropland maps (Monfreda hereafter; available at http://www.earthstat.org/). The researchers gathered statistics from the agricultural census data of 206 countries, classifying the data using the FAOSTAT classification system (Di Gregorio and Jansen 2000). Aggregating this data allowed the researchers to produce novel maps of eleven major crops, as well as maps on the crops’ net primary production. The work of Monfreda et al. (2008) is well accepted and used by numerous experts and integrated climate models, serving as the basis to harmonize global land use change from 850–2100 for CMIP6, for example (Hurtt et al. 2020).

### Data processing

The list of crops included in the tool represent the top crops globally, based on harvested area, net production value, and food supply (kcal/capita/day) for the year 2015, as reported by the Food and Agricultural Organization’s FAOstat database (http://www.fao.org/faostat/). For each category, we selected the top fifteen crops, excluding any items that represented crop groups or partial groups, as opposed to individual crops (i.e., “other vegetables” was excluded). By combining the top crops from each list (many of which overlapped), we were left with twenty-one crops that are important in terms of area, economic value, and/or food production. The total for these twenty-one crops represents just less than 75% of the total harvested area reported by Monfreda et al. (2008) (Supplemental Table S1).

We first calculated the total harvested area in each GEnS stratum and zone for each of the twenty-one crops. The GEnS data was aggregated to match the lower resolution of Monfreda data using a modal aggregate function. We then summed the area of each crop, as 1000 ha of harvested area, which fell within each stratum using a zonal function. All calculations were done in R using the “raster” package. We then converted the global scale, high-resolution GEnS raster data map tiles, which allows for storing and rendering high resolution maps in an efficient and easily zoomable way. Each GEnS zone was extracted and exported as a TIFF file from QGIS and then converted to tiles in Maptiler (https://www.maptiler.com/) using an x, y, z, folder structure at a zoomable scale of 0–10.

## Results

**Fig. 2:**
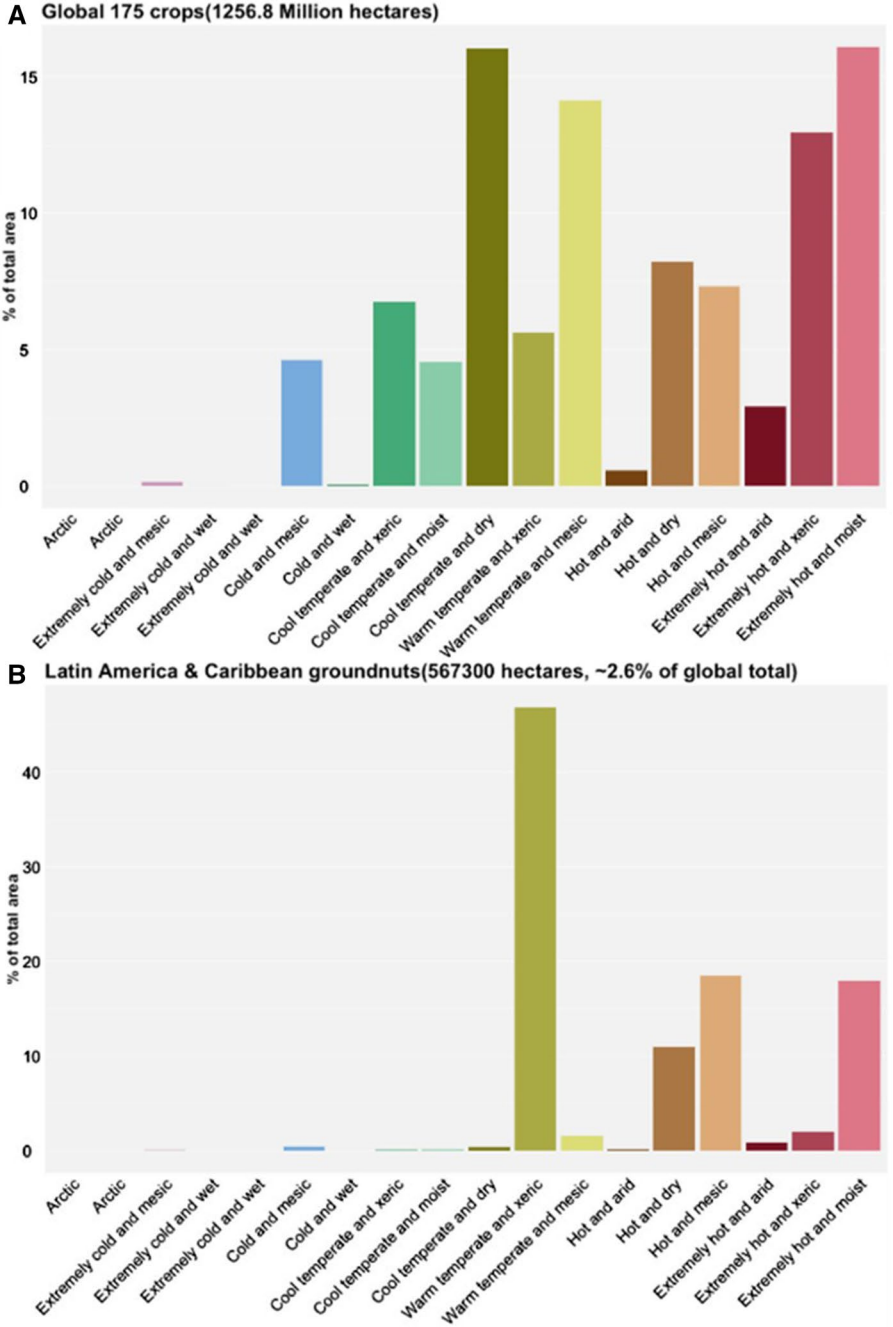
Histograms of the percentage of total area of the crops in each GEnZ as visualized in the GEnZ Explorer tool based on the GEnZ in which they are produced. **A** Shows the GEnZ of all 175 crops in Monfreda et al. (2008) and **B** shows the percentage of total area of groundnuts in Latin America and the Caribbean under each GEnZ, based on the data from Monfreda et al. (2008)

The combined data sets of the GEnS zonation of Metzger et al. (2013b) and Monfreda (2008) were used to create the Global Environmental Zones (GEnZ) Explorer, which is now a publicly available tool at https://foodsystems.org/resources/genz/. The tool allows users to: (a) generate crop histograms to visualize the GEnZ of a crop in a specific region by the total area of a crop, (b) view maps of crop distribution to visualize where selected crops are cultivated and the GEnZ at this location, and (c) use an interactive map to identify the GEnZ at specific locations. These are discussed in greater detail below. The Crop Histogram tool within the GEnZ Explorer allows users to see the percentage of total hectares of the crop produced in each of the GEnZ. This tool also provides users with the percentage of the total area for all production of the crop globally under each GEnZ as a histogram, as seen in Fig. 2A, or allows users to view areas of production for 21 specific crops at a global or regional scale, as seen in Fig. 2B, which summarizes the relevant extent of groundnut production in Latin America and the Caribbean in each of the GEnZ that co-occur with groundnuts in that region.

**Fig. 3:**
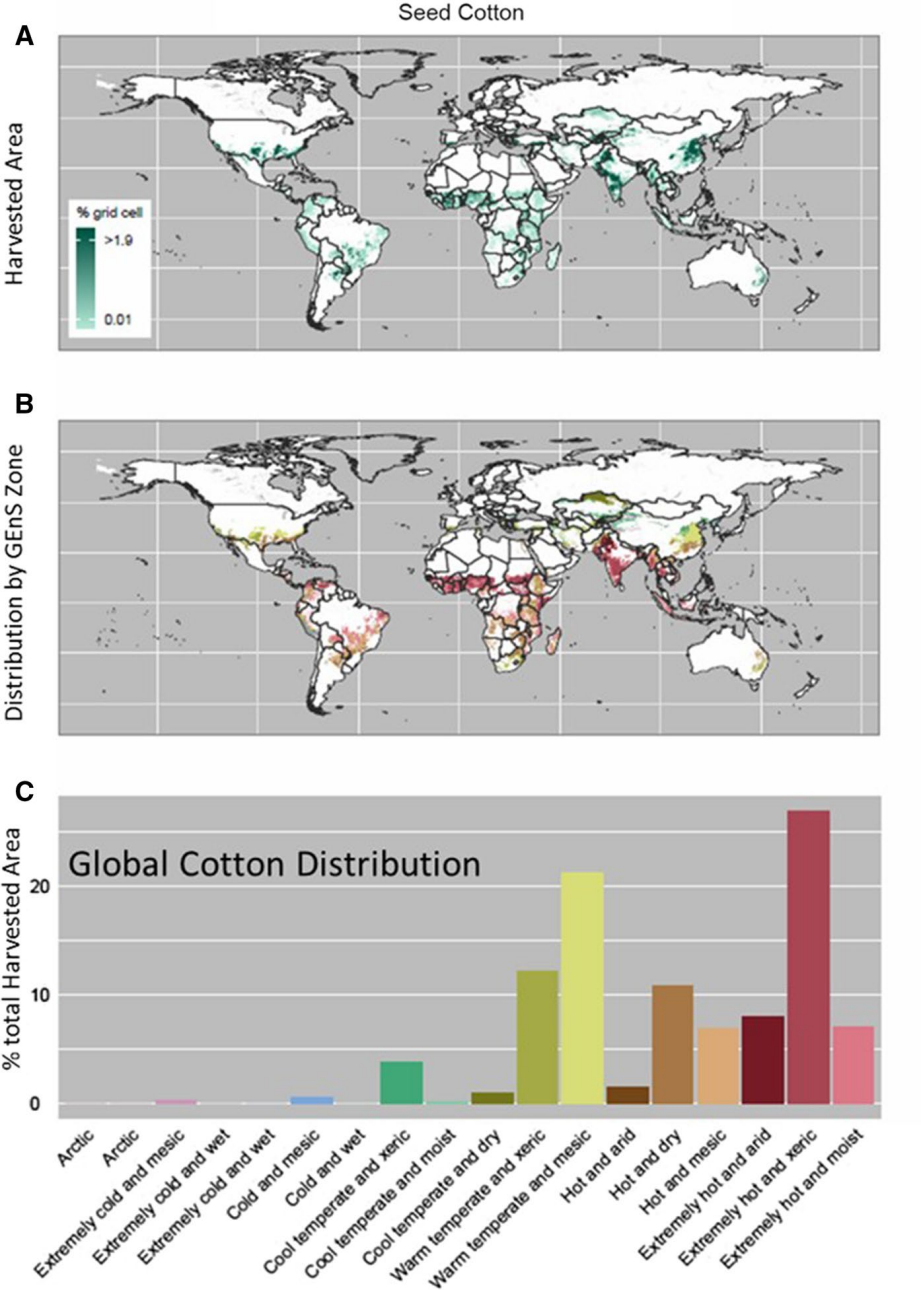
**A** Global cotton production (areas with dense green color indicate high cotton production). **B** GEnZ agroclimate zones overlaid on cotton production areas. **C** Relative distribution of global cotton production, as a percentage of total harvested area, each within GEnZ agroclimate zones. Bar charts include all values (no values are excluded from the calculation of totals in the histograms)

To demonstrate the utility of this tool, cotton was used as a case study example to determine the common areas in which cotton is grown. Figure 3A shows the global distribution (harvested area as a percentage of grid cell) of Monfreda data for cotton. Values under 0.0001 as a fraction of grid cell represents approximately 1 ha at the equator and are excluded from this plot. Maximum values were capped at the 90th percentile to show harvested area distribution more clearly. In Fig. 3B, a map of GEnZ zones was masked by harvested area (excluding all values under 0.0001 as a fraction of grid cell, as before), so only GEnZ data for grid cells in which the seed cotton is present are retained. This data is plotted to highlight the variation of zones in which seed cotton is present. This plot is strictly a qualitative representation of seed cotton by GEnZ. In combination, Fig. 3A and B provide a clear indication of the important GEnZ for seed cotton and where seed cotton production occurs globally (See Supplementary Information for data for other major crops).

Although not surprising, given the critical link between climate and plant productivity, the data indicate that commercial cultivation of cotton is limited to only a subset of agroclimatic zones throughout the world. Figure 3C shows the percentage of harvested area for cotton by GEnZ using the same color codes as Fig. 3B. One strength of this tool is the standardized presentation of the color of each GeNZ, whether on the map or histogram. Figure 3B and C together indicate the important zones for cotton, as well as the locations for crop production globally. There are seven principal agroclimatic zones for cotton production that account for more than 90% of total global cotton production (Fig. 3C). Most cotton production occurs in the Extremely Hot and Xeric agroclimatic zone (~ 28%), followed by the Warm Temperate and Mesic zone (~ 21%), the Warm Temperate and Xeric zone (~ 12%), and the Hot and Dry zone (~ 11%). The Extremely Hot and Xeric agroclimatic zone is extensive in both India and Africa, accounting for most of the cotton produced in these regions (Fig. 3C). Cotton production in the United States and China occurs primarily within the Warm Temperate and Mesic, Warm Temperate and Xeric, and Hot and Dry zones—three of the agroclimatic zones that both countries have in common (Fig. 3B). Specific relationships between crops and GEnZ are also apparent for the twenty-one additional crops analyzed (see Supplemental Table S1).

**Fig. 4:**
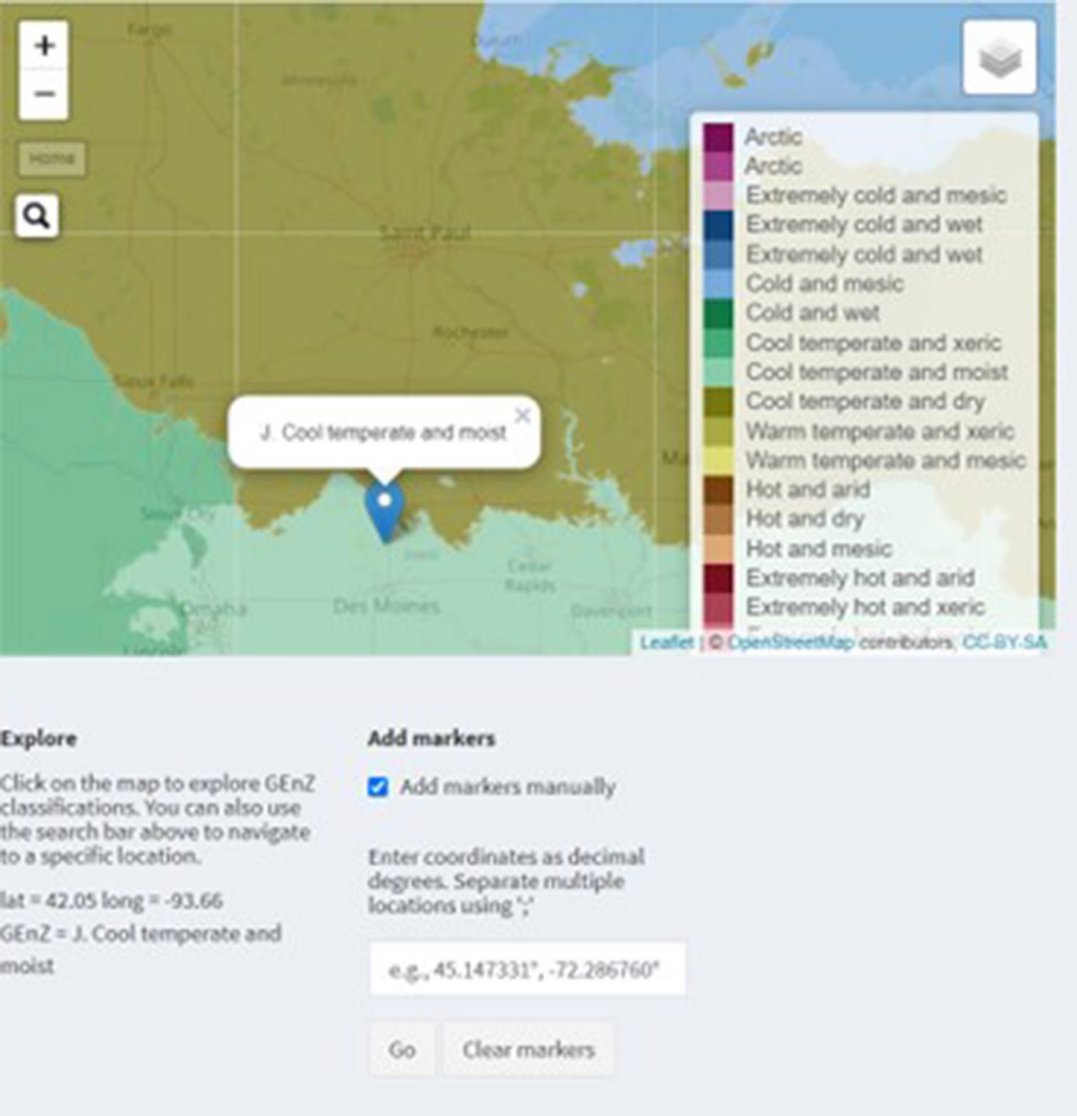
Screenshot of the GEnZ Map tool of the GEnZ Explorer, with a marker added for a specific location demonstrating that users can search by name or coordinates to determine the GEnZ at one or more locations

Lastly, the GEnZ Map tool in the GEnZ Explorer allows users to identify the GEnZ of a specific geographic location, as seen in Fig. 4. Users can manually place a marker at one or more locations on the map to determine the GEnZ of those locations. Users can place a marker by searching the name or address of a location (based on the Open Street Map Geocoder) or by providing the GPS coordinates of a location. In terms of data transportability, the GEnZ Explorer Map tool can identify the GEnZ of locations in which CFTs have already been completed, which can help evaluate whether they are suitable to inform an anticipated risk assessment. If new CFTs need to be established, researchers can use the tool to identify GEnZ at anticipated location(s) and then use the Crop Histogram tool to determine whether the anticipated locations are in GEnZ where production of that crop is common.

To describe how the GEnZ Explorer can be a useful tool to inform data transportability, the following discussion section of this paper has three specific case studies that demonstrate how the GEnZ Explorer can be used during the ERA process to facilitate the transportability of CFT data.

## Discussion

One example of the importance of agroclimate in informing data transportability was presented by Vesprini et al. (2020), which discusses Embrapa 5.1, a GE variety of common bean resistant to *Bean golden mosaic virus*. This variety was developed by Embrapa and CFTs were conducted in Brazil for the risk assessment prior to approval of cultivation. The authors proposed three criteria that would be useful for determining whether the data from the CFTs in Brazil could inform a risk assessment of Embrapa 5.1 in Argentina: (1) the CFTs were appropriately designed and used proper methodology, (2) the endpoints were relevant and consistent across studies, and (3) the environments of the crop production areas of the CFTs were relevant to the conditions in Argentina where cultivation is intended. Based upon their assessment, they determined that the previous CFTs would be useful in informing a risk assessment in Argentina. To assess the environmental factors, Vesprini et al. (2020) used historical information on environmental factors. Implementing GEnZ Explorer for this case, readily identifies the Brazilian CFTs to encompass agroclimate zones characteristic of production areas for common bean in Argentina.Below, we present three case studies to demonstrate how the GEnZ Explorer could contribute to rational decision making in the planning of CFTs and the use of CFT data for risk assessment.

### MON89034 Insect-resistant maize

**Fig. 5:**
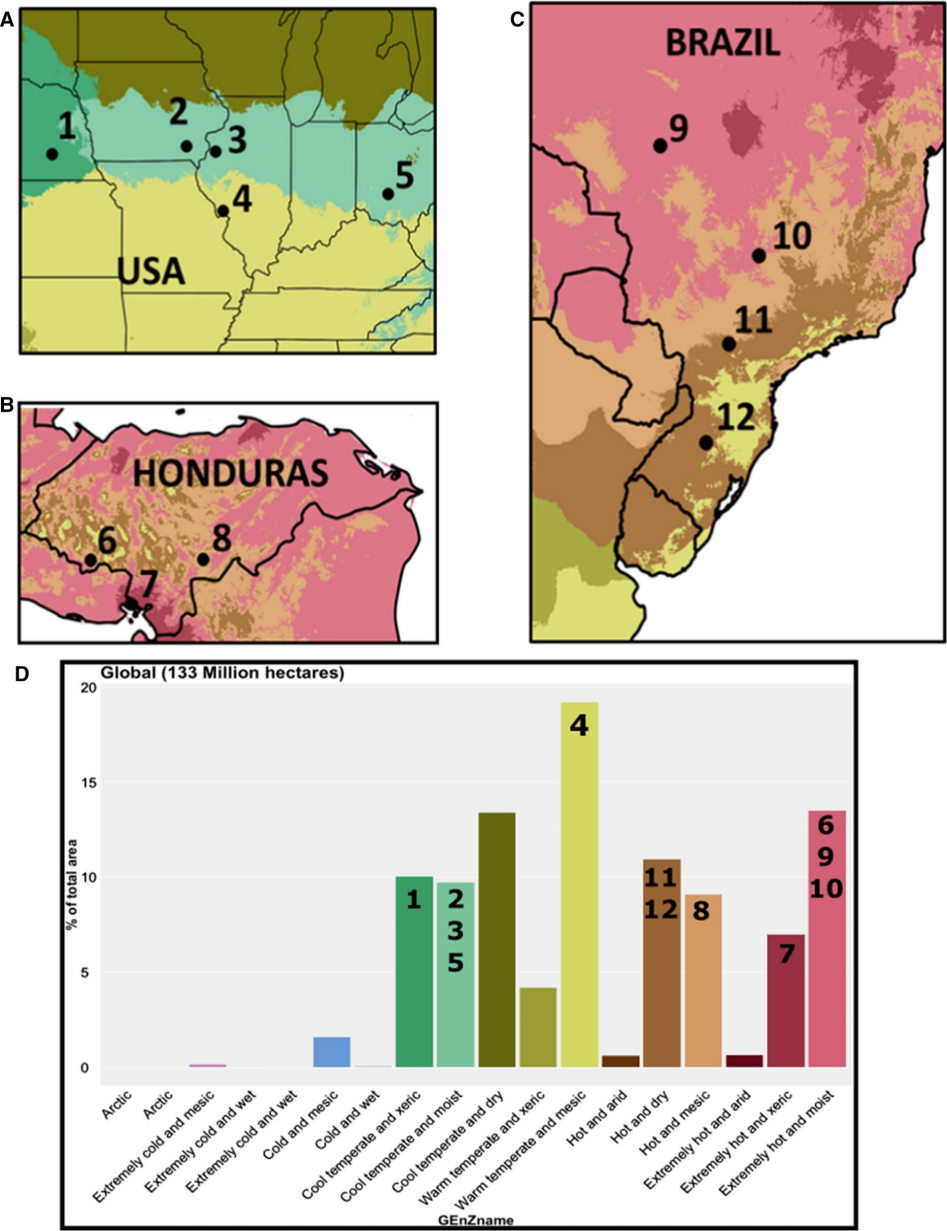
Locations and GEnZ of previous CFTs for MON89034 that were conducted for regulatory approval in Brazil, Honduras, and the United States. **A** CFT locations for MON89034 Bt maize in United States: 1. York County, NE 2. Jefferson County, IA, 3. Warren County, IL, 4. Jersey County, IL, 5. Fayette County, OH. **B** CFT locations for MON89034 Bt maize in Honduras: 6. Conception, 7. Playitas, and 8. Danli. **C** CFT locations for MON89034 Bt maize in Brazil: 9. Sorriso, MT, 10. Cachoeira Dorada, MG, 11. Rolândia, PR, and 12. Não-Me-Toque, RS. **D** Histogram of the percentage of the area for maize production globally by GEnZ, with the numbers of the CFT locations placed on top of the histogram

MON89034 is an insect-resistant maize that expresses two different Cry proteins from *Bacillus thuringiensis* subsp. *kumamotoensis* (Bt) to protect against damage from lepidopteran pests (EPA 2010). MON89034 (also known as YieldGard™ VT Pro™) provides a useful case study of a GE event that has been tested and approved for cultivation in multiple countries, which also has a history of safe use. CFTs for MON89034 Bt maize have been conducted in at least six GEnZ across nine countries (Table 2), including multiple locations in the United States, Honduras, and Brazil. Figure 5A–C present the GEnZ of these locations as visualized in the GEnZ Map tool of the GEnZ Explorer. There are numerous CFTs conducted across seven different agroclimatic zones to draw from for obtaining data to inform a new ERA. Across all these previous CFTs, no quantifiable differences have been measured, which indicates that comparison between GE and non-GE varieties is not influenced by agroclimatic conditions in any of those zones. Thus, testing more locations in the same GEnZ is highly unlikely to provide additional information for ERAs. It also suggests that the CFT data already collected from these zones have value for helping to satisfy the requirement for local CFT data in risk assessments for MON89034 for most maize-producing countries worldwide.

**Table 2:** List of countries that have approved the cultivation of maize variety MON89034 transformed with the cry1A.105 and cry2AB2 insecticidal proteins from *Bacillus thuringiensis* subsp. kumamotoensis, according to the GM Approval Database at isaaa.org as of the most recent update on September 2, 2021

Countries with MON89034 approved for cultivation
Argentina
Brazil
Canada
Honduras
Japan
Paraguay
Philippines
South Africa
United States
Vietnam*

Using the tools in the GEnZ Explorer allows researchers and risk assessors to determine the agroclimatic zones of the previous CFTs in Brazil, Honduras, and the United States, which are mapped on top of the histogram of GEnZ and percent total harvested area in Fig. 5D. As is shown here, CFT trials from the three countries would provide data from nearly all of the major GEnZ for maize production, except for Cool Temperate and Dry. If a new country was conducting a risk assessment for cultivation of MON89034 Bt maize and had production in the Warm Temperate and Mesic, Hot and Dry, or Hot and Mesic GEnZ in their country, then the risk assessors could use the data obtained at the previous field trials in Jersey County, IL, United States (location 4), Danli, Honduras (location 8), and Rolândia, PR and Não-Me-Toque, RS, Brazil (locations 11, 12) to inform the ERA, and they might decide additional CFTs in the GEnZ are not necessary. In some countries, regulatory authorities require that new CFTs be conducted regardless of whether relevant data already exists or not. For those countries that always require new trials, the risk assessors could use their knowledge of the previous CFT locations to best place the new CFT in GEnZ where the additional data would add value (e.g., if the country has regions of maize production that is in the Cool Temperate and Dry GEnZ), as this might allow the researchers to generate data in an untested GEnZ).

The potential value of data transportability is demonstrated within the context of the present threat to maize production in Africa from fall armyworm (*Spodoptera frugiperda*), as MON89034 Bt maize is effective against fall armyworm. The recent introduction of this pest in Central and Western African in 2016 (Goergen et al. 2016) and subsequent spread across the continent has resulted in extensive crop loss throughout Sub-Saharan Africa (FAO et al. 2018). Uganda, Kenya, and Malawi all suffered major losses in maize production as a result of fall armyworm in 2017, resulting in a 25–40% reduction in crop yield (Day et al. 2017). Research field trials conducted in Kenya through the TELA maize project showed that drought and insect-resistant maize, including MON89034 Bt maize, are valuable tools to combat these stressors in the region and has led to the seeking of approval for MON8904 maize to be cultivated in the country. The data gathered in these field trials and others across the globe could inform the risk assessments of MON89034 Bt maize that are being sought in countries across the continent.

The GEnZ Explorer could provide regulators with the necessary tools to assist with data transportability, which would expedite the process of getting effective insect resistant varieties to producers. The tool would be useful in allowing data transportability for African nations beginning to consider regulatory approval of Bt maize and could assist with the regional harmonization of biosafety reviews currently proposed by the Common Market for Eastern and Southern Africa (COMESA), which is composed of 21 member states (Akinbo et al. 2021). *For example, the Kenyan national performance trial on Bt maize were conducted in five locations with GEnZ of hot and mesic (Alupe, Kibos, Mwra) and hot and dry (Embu, Kakamega, Thika). These locations were similar GEnZ to previous trials in Honduras and Brazil. CFTs for MON89034 Bt maize and stacked maize in Nigeria were conducted in extremely hot and moist GEnZ, which was similar to one location in Honduras and two in Brazil.* With the increasing and continuous spread of new and emergent pests across the globe, this example highlights how data transportability can facilitate regulatory approvals for events that address an immediate need and reduce the time required to affect varieties to producers.

### Virus-resistant cassava (VIRCA)

A second data transportability case study involves a GE event that is nearing cultivation in Western Africa. Cassava is the second most important staple food crop in Africa. For nearly 70 years, cassava crops have suffered important economic losses from cassava brown streak disease (CBSD), a viral disease endemic to coastal cassava growing regions in Western Africa and has been spreading into new regions. The challenge with managing the disease has been that African cassava varieties lack natural resistance, and there is general paucity of agrochemicals to treat viral diseases, leaving African farmers to manage the impact of the disease by early harvesting.

**Fig. 6:**
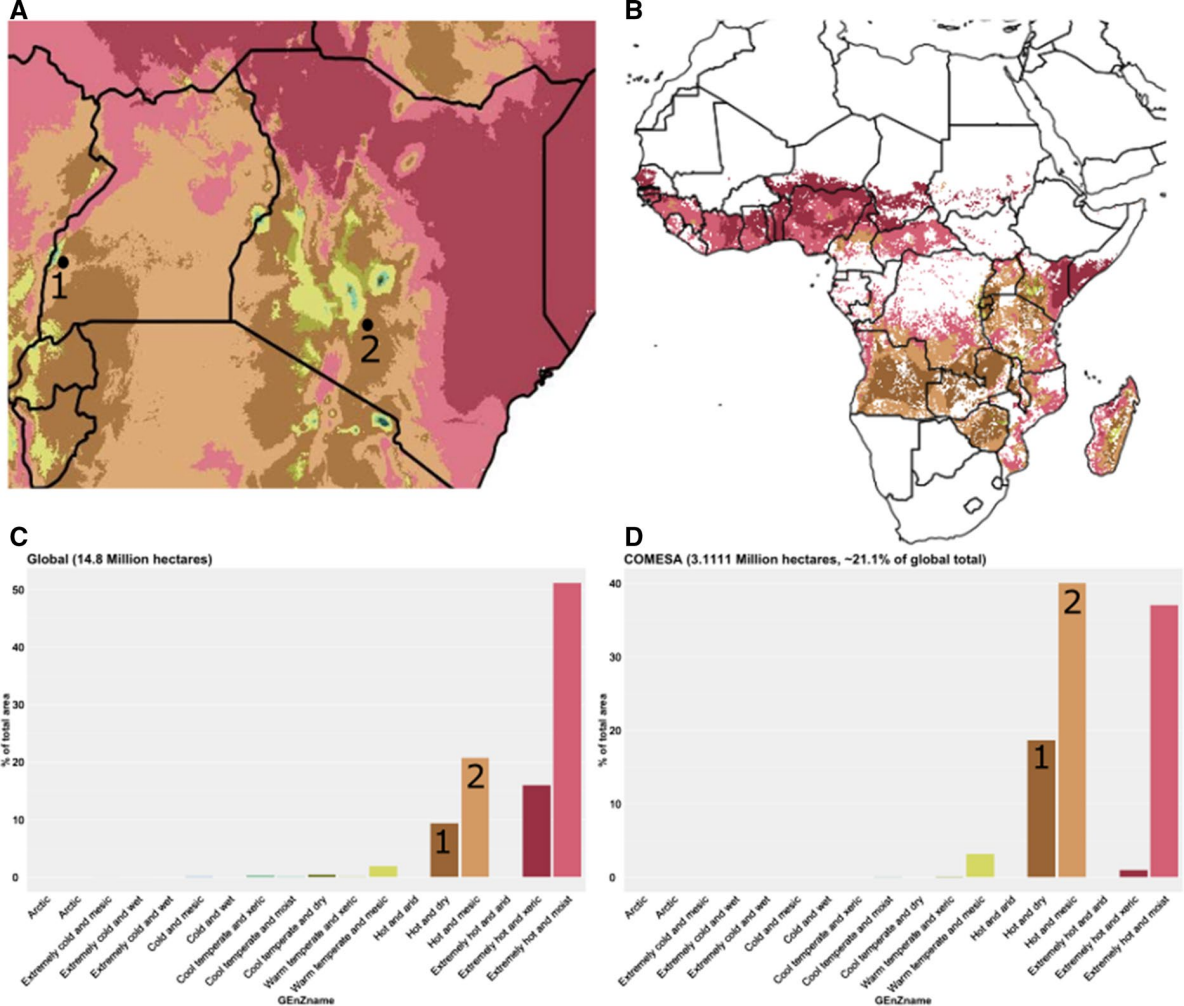
**A** Locations of virus-resistant cassava confined field trials in Uganda and Kenya as placed on a map with GEnZ coloration. The black dot with 1 is Kasese, Uganda, and the one with 2 is Kandara, Kenya. **B** Distribution of cassava crop production in Africa displayed within GEnZ agroclimate zones. **C** Histogram of the percentage of the area for cassava production by GEnZ globally, with the numbers of the CFT locations placed on top of the histogram. **D** Histogram of the percentage of the area for cassava production by GEnZ in COMESA member states, with the numbers of the CFT locations placed on top of the histogram

The VIRCA Plus project developed a GE cassava highly resistant to both viruses the cause CBSD (Beyene et al. 2017; Wagaba et al. 2017), which can greatly reduce producer losses. CFTs with the VIRCA-improved cassava have been conducted in Kandara, Kenya (corresponding to the Hot and Mesic zone) and Kasese, Uganda (corresponding to the Hot and Dry zone). CFTs were also conducted in Rwanda in 2021. The locations of these CFTs are indicated as black circles on the map in Fig. 6A. These zones are shared by cassava producing countries in the region, such as Burundi, Ethiopia, Rwanda, Madagascar, Zambia, and Zimbabwe, as seen in Fig. 6B. While globally, most cassava is grown in extremely Hot and Moist environments (Fig. 6C), the most prevalent GEnZ in the COMESA countries (Fig. 6D), where CBSD is endemic, is Hot and Mesic, which is the same GEnZ as the CFT in Kandara, Kenya.

The two CFTs conducted in Kenya and Uganda were in the first and third most common GEnZ for cassava production in COMESA, at 40% and 18%, respectively (Fig. 6D). These trials could therefore provide useful information as part of the risk assessment process for this event for a considerable number of countries throughout eastern and southern Africa. In particular, a large area of Rwanda is Hot and Dry, similar to Kasese, Uganda. In June 2021, the Kenyan National Biosafety Authority (NBA) approved the environmental release of VIRCA cassava after considering data from CFTs in both Kenya and Uganda. Use of data generated in Uganda in the Kenyan risk assessment indicates that regulatory authorities are open to data transportability, which could help streamline the risk assessment process. Due to the limited resources available to public sector developers, such as those developing VIRCA cassava, the locations of CFT trials can be strategically selected to maximize the usefulness of the data. As 36% of cassava in COMESA member states and over 50% of cassava globally is grown at locations with extremely Hot and Moist GEnZ (Fig. 6D), scientists setting up future CFTs might want to target locations with this GEnZ, as previous trials have been conducted in the other two predominant GEnZ for cassava.

### Golden rice

Rice is a major staple, but reliance on rice as a primary food source in food insecure populations has led to serious health problems due to vitamin A deficiency (Tiozon et al. 2021). The WHO estimates that 33% of pre-school aged children have vitamin A deficiency. To address this public health concern, researchers have developed a GE rice that produces β-carotene, called GR2E rice or more commonly known as Golden Rice (Swamy et al. 2019). This improved rice could help to alleviate Vitamin A deficiency for regions where rice is the predominant staple food. Golden Rice has been approved for food use in Australia, Canada, and New Zealand and for food and feed use in the United States and the Philippines (Owens 2018; Oliva et al. 2020). The Philippines approved Golden Rice for commercial cultivation in July 2021.

**Fig. 7:**
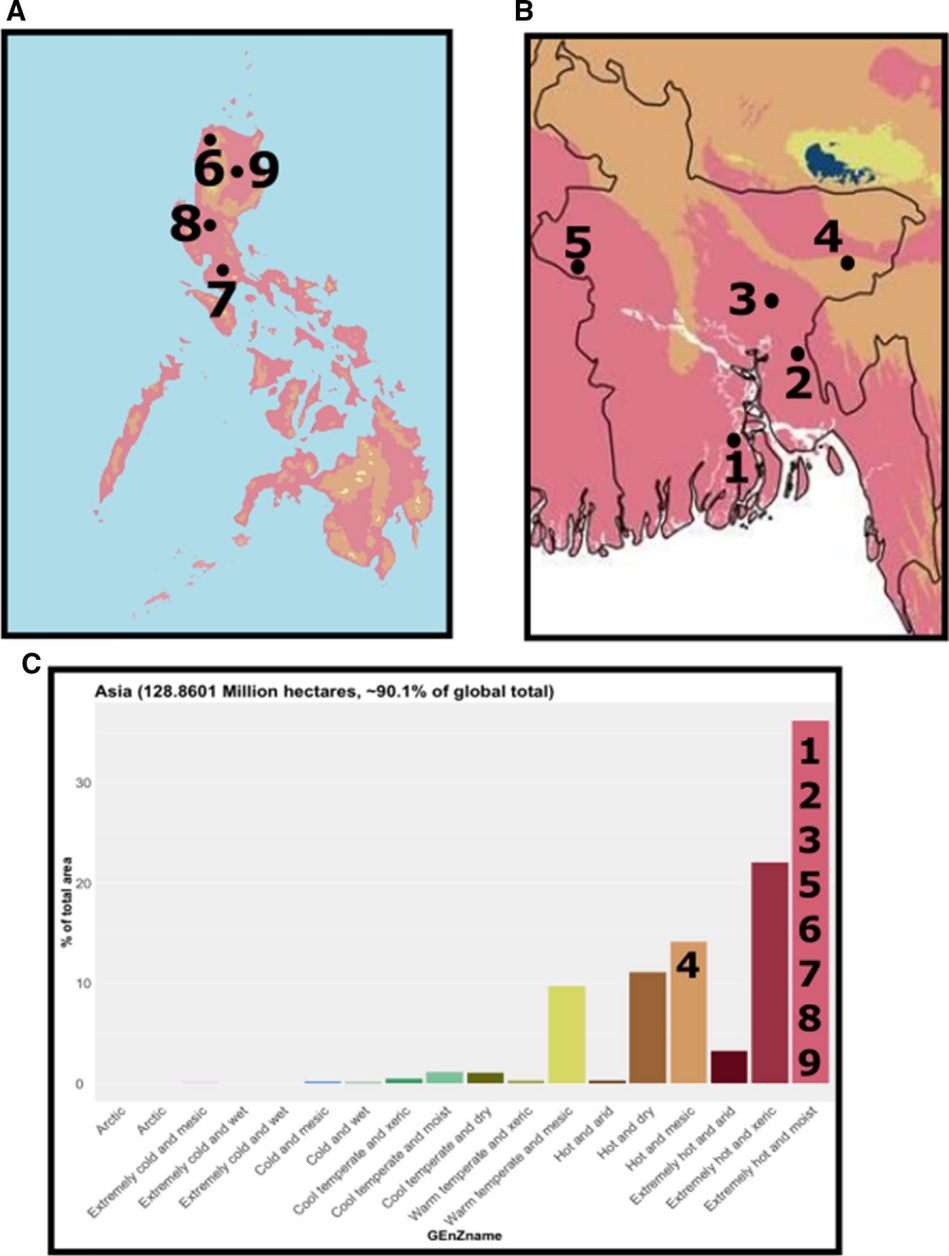
GEnZ of rice production in Asia. **A** Locations of CFTs for Golden Rice in the Philippines, placed on a map with GEnZ coloration. **B** Location of CFTs for Golden Rice in Bangladesh, placed on a map with GEnZ coloration. **C** Histogram of the percentage of the area for rice production in Asia by GEnZ, with the numbers of the CFT locations placed on top of the histogram

To inform cultivation, CFTs for Golden Rice have been conducted in the Philippines (Mallikarjuna Swamy et al. 2021) and Bangladesh (Biswas et al. 2021). The International Rice Research Institute (IRRI) conducted four CFTs at IRRI and PhilRice in two consecutive years (Fig. 7A, http://biotech.da.gov.ph/upload/GR2E/GR2E-FFP-supporting-dossier-PH.pdf). The Bangladesh Rice Research Institute (BRRI) conducted CFTs at five sites, at BRRI research stations across the country (Fig. 7B). The GEnZ of all these locations, except one, is Extremely Hot and Moist, which is the most common GEnZ for production of rice both globally and in Asia. The remaining trial was in the Hot and Mesic GEnZ (Fig. 7C). In all of these trials, the GR2E transformed rice does not have significant differences in terms of agronomic performance or yield compared to the untransformed isoline (Biswas et al. 2021; Mallikarjuna Swamy et al. 2021).

Eight of the CFTs for Golden Rice were tested in the Extremely Hot and Moist GEnZ, where over 35% of rice is grown, while one was in the Hot and Mesic GEnZ, where 14% of rice is grown (Fig. 7D). The GEnZ Explorer tool could be useful in informing site selection for future CFTs, as there have yet to be trials in the Extremely Hot and Xeric GEnZ, which is the second largest area under rice cultivation. Additionally, the data from the existing CFTs in Bangladesh and the Philippines could be used to inform future regulatory considerations for the cultivation of Golden Rice in Asia. As Golden Rice is intended to help food insecure children with Vitamin A deficiency, being able to streamline the regulatory review could shorten the time to cultivation and provide an option for reducing a leading cause of childhood blindness in areas where rice is the staple, such as during future considerations in India, Indonesia, or elsewhere.

## Conclusions

Given that agroclimate has been identified as the primary differentiating factor between locations where CFTs are conducted, a comparison between the GE crop and non-GE crop within an agroclimatic zone is expected to yield the same results regardless of the country in which the trial is conducted. Performing additional CFTs within the same agroclimatic zone are unlikely to provide additional useful data for the risk assessment. The GEnS zonation (Metzger et al. 2013a, b) provides a means for regulators to meet their need to provide scientific justification demonstrating the relevance of existing CFT data to inform an ERA by classifying the agroclimatic conditions associated with CFTs conducted outside of their country. Combining this data with the cropping data of Monfreda et al. (2008) in the GEnZ Explorer tool allows users to determine the common agroclimates in which a crop is produced. As the GEnZ are based solely on measurable agroclimatic parameters and well accepted cropping data, they provide a scientific basis for defining agroclimatic zones that is reproducible, publicly available, and without subjective bias.

The GEnZ Explorer (https://foodsystems.org/resources/genz/) is a tool that can aid data transportability for the ERAs of crop biotechnology. Other rationales have been provided to further support the use of data transportability to allow regulators to use data generated in previous CFTs in other regions, such as familiarity (Capalbo et al. 2020) and simplification of regulatory procedures (Benítez Candia et al. 2020). Others agree that an understanding of data transportability is important for the readiness of regulatory systems in countries preparing to start the regulatory approval process for GE crops (Zawedde et al. 2018). Using data generated from other trials could help to streamline approvals for events that have a history of safe use in other countries, such as Bt maize. Tools such as the GEnZ Explorer, which ease the use of data transportability, could help regulatory authorities, as regulators spend money to oversee confined field trials, review field reports, etc., and it simply may not be cost effective to replicate trials for an event that is already approved for cultivation in several other countries. Using data from previous CFTs can reduce repeating CFTs in the same GEnZ, but in different countries, which can in turn save regulators time and money.

Additionally, the GEnZ Explorer can provide a scientific basis for regulatory authorities looking to use data transportability to expedite the review process if there is an imminent national interest, such as an emergent pest, when effective varieties have already been tested and commercialized elsewhere. As demonstrated in the case studies, a tool that could assist in the transportability of data to inform an ERA has the potential to reduce the regulatory review time and subsequently, the time required to get desired varieties to producers. The GEnZ Explorer can encourage data transportability to assist with getting regulatory approvals in countries where the market might not warrant the cost of expensive new CFTs, such as areas with low production of a crop or those with subsistence producers. Transporting previous CFT data to inform an ERA can also benefit farmers by potentially reducing the time for regulatory approval, providing access to varieties that can help manage emergent pests and conditions, such as the emergency resulting from the spread of fall armyworm across Africa and Asia in recent years.

In the cases of VIRCA and Golden Rice, which were created by public sector developers, using the GEnZ Explorer has the potential to help inform whether the data from existing CFTs could be used to inform risk assessments in other countries. As both VIRCA cassava and Golden Rice are products of public sector developers, the cost of replicating CFTs in each country in the same GEnZ is likely cost prohibitive to providing access to this technology. Multiple replications of trials in the same GEnZ in different countries is expensive due to the cost associated with finding and establishing locations, training people to complete trials, etc. However, if using the data from previous trials is possible, it would reduce the burden not only to the developer, but also to the regulatory authorities who must oversee the trials. If a regulatory authority still requires a CFT in the country or the if the past CFTs are not in the same GEnZ, the GEnZ Explorer can still inform public sector developers, as they can use the tool to identify common GEnZ for the production of a crop. Kenyan authorities using data from Ugandan trials for VIRCA cassava in their decision-making process sets a precedent for transportability of data to other African countries. The GEnZ Explorer can help inform regulators about whether trials were completed in Kenya and Uganda, and current CFTs in Rwanda can be used to inform future risk assessments. Beyond the benefit to public developers with limited funding, streamlining the regulatory approvals of varieties approved in other countries can directly benefit farmers by reducing the time for approving such varieties.

## Supplementary Information

Below is the link to the electronic supplementary material.

Supplementary file1 (DOCX 12 kb)

## Data Availability

The global climate classification used in this web tool is the Global Environmental Zones (GEnZ) produced by Metzger et al. (2013b). Crop distribution data is from Monfreda et al. (2008). The GEnZ Explorer tool is available to anyone at no cost by visiting https://foodsystems.org/resources/genz/ and creating a free account.
