# Supplementary material for: GEnZ explorer: a tool for visualizing agroclimate to inform research and regulatory risk assessment
Source: Transgenic Res. 2023 Jun 6;32(4):321–37. doi: 10.1007/s11248-023-00354-w (PMC10409678; doi:10.1007/s11248-023-00354-w)
Supplement: Supplementary file 1 — Supplementary file1 (DOCX 12 kb) [file 11248_2023_354_MOESM1_ESM.docx]

**Supplemental Table 1: Listing of the 21 crops that represent just less than 75% of the total harvested area reported by Monfreda et al. (2008) and are the focus of the GEnZ tool.**

| Apple |
| --- |
| Banana |
| Barley |
| Beans |
| Cassava |
| Cotton |
| Grape |
| Groundnut |
| Maize |
| Mango |
| Millet |
| Oil Palm |
| Potato |
| Rapeseed |
| Rice |
| Sorghum |
| Soybean |
| Sugarcane |
| Sunflower |
| Tomato |
| Wheat |
